# Ectoparasites of Camels *(Camelus dromedaries)* in Afar Pastoral Areas of Ethiopia

**DOI:** 10.1155/vmi/5550074

**Published:** 2025-04-11

**Authors:** Angesom Hadush Desta

**Affiliations:** Department of Animal Sciences, College of Agriculture, Aksum University Shire Campus, Shire, Ethiopia

**Keywords:** lice, mange, nasal fly, productivity, tick

## Abstract

Camel external parasites are important health problems that have the potential to affect camel welfare and productivity. A cross-sectional study using ectoparasitic examination and key informant interviews was done in camels (*Camelus dromedarius*) in some districts of Afar region, Northeast Ethiopia, to investigate major external parasites and to collect the required information. A total of 384 camels were examined for the presence of any external parasite and 368 (95.8%) of them were infested at least with one of the different genera of ticks, sucking lice, Sarcoptes mange, and a camel nasal fly called *Cephalopina titillator*. Tick infestation (89.1%) followed by mange (36.7%) were the main ectoparasites found in the study area. Mixed infestations of ticks with mange (30.2%) and ticks with lice (19.8%) were higher than the other types of camel ectoparasite infestation. Around 1424 male and female ticks were collected and identified as the genera of *Amblyomma*, *Hyalomma*, *Rhipicephalus*, and *Boophilus*. A higher incidence and spread of external parasites have occurred in areas having cross-border salt trade by camels and mixing of herds at the feeding and watering points. Hence, education on modern camel husbandry and management to the camel owners and regular antiparasitic treatment and preventive measures has to be conducted continuously.

## 1. Introduction

The dromedary camels (*Camelus dromedarius*) are one-humped camels which are versatile species of animals well made to order fluctuating climatic conditions and feed problems of dry areas [1, 2]. These species own astonishing adaptation mechanisms that make them to continue alive and produce in unfriendly environment [3]. Dromedary camels are resourceful existing assets that help to guarantee livelihood by serving as a source of food mainly milk and meat, material packaging and transporting, sociocultural contributions such as social gifts, family reputation, and reimbursement [4, 5]. Around 80% of the world's dromedary population is found in Africa and 63% of it ascribed to Horn of Africa [6].

In Ethiopia, dromedary camels are found in the arid as well as semiarid zones that represent above 60% land coverage with millions of pastoral and agropastoral societies which include Afar, Borena, and Somalia, where camel rearing is their major activity [1, 7]. The foremost means of livelihood in these areas is explicitly camel production [1, 8].

Despite camels have such an enormous contribution to the community living in dry, difficult, and environmentally unfriendly areas, its full exploitation is challenged by diseases, specifically parasitic diseases, lack of feed, traditional husbandry practices, and poor veterinary services [2, 9]. Among camel diseases, external parasites constitute significant health challenge that affects the maximum production and performance of camels [5].

Camel external parasites such as ticks, manges, and lice are of prime magnitude to hamper the maximum utilization and productivity of these animals [10]. In east Africa, the mange species, *Sarcoptes scabiei var cameli*, is a small burrowing mite that affects infantile and immune-compromised adult that build up a chronic form in camels [11, 12]. A nasal larva of a fly called *Cephalopina titillator* is another parasite that causes nuisance and frustration in camels which is commonly prevalent in pastoral areas [13]. The larvae are usually appreciated while the animal sneezes because of the irritation it creates inside the nasal cavity, which can be complicated with secondary bacterial infections [14].

In Ethiopia, there are reports of camel ectoparasites such as ticks, lice, and nasal bot [10] and mange [15] in the predominantly camel-rearing drier areas of the country [16]. Conveniently, there is a need to investigate further the type and incidence of external parasites in dromedary camels in the uncovered pastoral areas of Ethiopia. Hence, this study was initiated to identify and determine the prevalence, types of parasites, and seasonal occurrence of the major external parasites of camels in the Afar pastoral area of Ethiopia from December 2020 upto June 2021.

## 2. Materials and Methods

### 2.1. Description of Study Areas

The study was done in the Afar regional state of Ethiopia, part of the Great Rift Valley with elevations ranging from around 116 m below the sea level to approximately 2063 m above the sea level, covering the pastoral areas of northeastern of the country. It is located in a geographical latitude of 11°48′59.99″N and a longitude of 41°24′59.99″E. The area has a varying temperature of 25°C during the rainy season and 48°C during the dry season. The climate is characterized as arid and semiarid range land with low and erratic rainfall having an average annual rainfall of 500 mm in the semiarid western escarpments, decreasing to 150 mm in the arid zones to the east [17]. The major means of income in the area is animal production with a livestock population of over 10 million of which around 859,580 (8.5%) are dromedary camels [18]. The regional state has five administrative zones divided into 32 districts with pastoral and agropastoral systems as the main types of livelihood experienced [19]. Three study districts were purposively selected from two zones, namely, Aba'ala and Berhale of Kilebeti Resu and Dubti district of Awsi Resu Zones.

### 2.2. Study Population

The study was done among the camel populations of Afar breed under extensive production system in Aba'ala, Berhale, and Dubti districts with a population of around 28,834, 13,366, and 5966, respectively [18]. The camels were of both sexes and classification of age was done as young (6 months up to 4 years) and adults (above 4 years) [6, 20]. The body condition of camels was also scored from 5 (excellent) to 0 (very poor) according to the characteristics established by Faye and Bengoumi [21]. Furthermore, a key informant interview was done with animal health professionals, camel herders, and salt merchants to supplement the findings.

### 2.3. Study Design

A cross-sectional type of study was done in Afar dromedary camels (*Camelus dromedaries)* to identifying external parasites and associated factors in the Afar pastoral area of Ethiopia from December 2020 upto June 2021.

### 2.4. Sample Size and Sampling Methods

Three districts, namely, Aba'ala, Berhale, and Dubti districts from two zones were selected purposively based on the availability of cross-border camel traveling among the regions, salt trade using camels, and the presence of abundant population of camels. The lowest administrative unit at district level is Pastoral Association/PAs and there are 11 PAs in Aba'ala, 9 PAs in Berhale, and 15 PAs in Dubti district. In each selected district, around 30% of the PAs available were considered representative to the sampling areas based on affordability. Therefore, 4 PAs from Aba'ala, 3 PAs from Berhale, and 5 PAs from Dubti were randomly selected.

As there was no earlier report on camel external parasites in the study districts, the standard predictable prevalence is assumed to be 50% within the 95% confidence interval (CI) at 5% desired accuracy. Consequently, a formula suggested by Thrusfield [22] is used to calculate the sample size (*n*) as follows:

(1) $$\begin{array}{c} n=\frac{{\left(1.96\right)}^{2}\times P_{\text{ex}}\times \left(1-P_{\text{ex}}\right)}{d^{2}}, \end{array}$$

where *n* = sample size, *d* = desired absolute precision (0.05), and *P*_ex_ = expected prevalence (50%), thus the desired total sample size for *P*_ex_ = 0.5 is *n* = 384.

Based on the proportional allocation of sample size to study districts considering the total camel population available in each study, around 230, 106, and 48 samples were collected from Aba'ala, Berhale, and Dubti districts, respectively. Demeke [23] has reported an average herd size of around 14 camels in the pastoral areas of Ethiopia; accordingly, the total sample size which is 384 were collected from 56 herds (384/14 ≈ 28 herds) or (clusters) and doubled to 56 clusters to increase accuracy. Herd size allocation was made proportionally to the three study districts and the herd sizes that required total samples randomly collected were about 34, 15, and 7 camel herds from Aba'ala, Berhale, and Dubti districts, respectively [22].

Individual camels from each randomly selected herd were proportionally sampled based on the number of camels found in that herd using the systematic random sampling technique on the spot [19]. In addition, a key informant interviews was done with 112 respondent camel owners and salt merchants and about 37 animal health professionals and experts.

### 2.5. Study Methodology and Data Collection

#### 2.5.1. Ectoparasites Examination

Examination of each selected study camel was done by visual inspection for any type of external parasites and palpation of skin lesions. For those positive camels on clinical examination, a detailed history was taken from the owner. Subsequently, ticks, lice, nasal fly, and skin scrapings were taken from each camel after proper restraining. Tick samples were collected carefully by rotating and in a horizontal pull to the body surface to avoid damage to the tick's mouth parts and preserved in a labeled plastic container with 70% ethanol. The collected ticks were observed under stereo microscope to identify up to species level following the guideline provided by Walker and his colleagues [24]. Based on this guideline, the key features used for identification included the scutum, anal groove, festoon (ornamentation), color, size, shape of mouthparts, and color of the legs.

Lice were collected from accessible body parts of the camels, mainly head, neck, and withers and preserved similarly to ticks. The nasal cavity of camels was also examined for the presence of nasal flies. Skin scrapings were collected and preserved in a container with 10% formalin and taken to laboratory. In order to release the mite from scabs and crusts, 10% KOH was added to the specimen before examination [25] and the species level identification was made according to the characteristics provided by Urquhart and his colleagues [26]. The samples were collected both in dry and rainy seasons [19]. While taking the samples, a format was prepared to record individual camel's history including sex, age, body condition score, and other characteristics.

#### 2.5.2. Key Informant Interviews

Camel owners from each sampled herd and interested salt merchants who use camels for their work are interviewed as key informants by raising questions such as husbandry and management practices, risk factors for transmission of ectoparasites among camels, and the indigenous treatment methods used. Furthermore, available animal health professionals were questioned for name of the common external parasites in the areas, possible means of transmission and the control and prevention of these parasites.

#### 2.5.3. Data Management and Statistical Analysis

The data collected were cleaned and stored in Microsoft Excel 2010 spread sheet after coding and SPSS Version 20 was used for analysis. Using this software, descriptive and analytical types of statistical computations were done to calculate frequency, prevalence, ratio, and to make comparison for infestation intensity and relative prevalence among the examined parasites [22]. The equations used are as follows [27]:

• Infestation prevalence (%) = 100⁣^∗^ (number of infested camels/number of examined camels).
• Tick infestation intensity = the number of ticks/the number of infected camels.
• Sex ratio = the number of female ticks to the number of male ticks.
• Relative prevalence = 100⁣^∗^ (number of tick species/the total number of ticks).

In addition, the association of risk factors with parasitic infestation was measured using multivariable Logistic regression analysis and odds ratio (OR) of 95% CI was computed to see the degree of association [22].

## 3. Results

A total of 384 camels were examined for the presence of any type of external parasite and 368 (95.8%) of them were infested at least with one external parasite. Based on the statistical logistic regression analysis, all risk factors had no significant association (*p* > 0.05) with ectoparasitisim (Table 1).

The major ectoparasites identified were different genera of ticks, sucking lice, *Sarcoptes mange*, and a camel nasal fly called *Cephalopina titillator*. Tick infestation (89.1%) followed by mange (36.7%) were the main ectoparasites found in the study area (Table 2). Mixed infestations of ticks with mange (30.2%) and ticks with lice (19.8%) were higher than the other types of camel ectoparasite infestation (Table 3).

Around 1424 male and female ticks were collected from the body of the camels and the tick genera identified were *Amblyomma, Hyalomma, Rhipicephalus, and Boophilus* (Table 4). The species level characterization with its infestation intensity of each identified tick parasite is also presented in Table 5.

### 3.1. Key Informant Interviews

In the key informant interviews, a total of 112 owners and salt merchants of camels and about 37 animal health experts have participated. From the 112 camel owners and salt merchants interviewed, around 98 (87.5%) were male and 14 (12.5%) were female and only 26 (23.2%) were literate. Among the 37 animal health professionals, only 9 (24.3%) were female professionals.

Accordingly, 100% of the respondents believed that external parasitism is the first priority to hamper the health and productivity of camels in the pastoral areas. The animal health experts thought that these parasites challenge camel productivity and welfare because it causes stressful conditions, which can be complicated with other diseases in camels especially on she camels.

Around 79% of the respondents said that the risk factor for the occurrence and spread of parasitic infestation were continuous contact among camels coming from different areas in the cross-regional salt trade streak between adjacent regions mainly around Berahle and sometimes in Aba'ala districts. All respondents (100%) said that mixing during feed and water search in common field areas, recurrent drought, poor managing, and husbandry practices are other factors that contributed to the spread of ectoparasites among camels and between herds. They also described that camels affected by these external parasites; they got damaged by other infections and diseases.

All animal health experts interviewed (100%) complained that the major external parasites in the study area were ticks and mange. Especially those professionals in Aba'ala and Berahle (82%) told that the prevalence of mange in these areas is expected to be high because of its contagious nature and the presence of cross region salt trade using camels. This cross-region salt trade using camels has been facilitating the transmission of all contagious diseases including manges and ticks due to mixing of camels coming from different areas. Those professionals in Dubti district (18%) told that mixing of camels of different herds at watering and feeding points has facilitated the transmission of ectoparasites among camels.

All animal health professionals interviewed (100%) said that the only commonly used antiectoparasitic drugs are ivermectin and diazinon spray in majority veterinary clinics. These showed that it is not only the shortage of types of drugs but also the quantity of drugs that has created problems in the treatment and control of camel external parasites. All camel owners (100%) said that they use traditional methods to treat their camels, particularly applying mud, leaf, and roots of plants, dung, etc. on the body of their camels to treat external parasites such as ticks, lice, and mange. Moreover, all respondents (100%) identified that sarcoptes mange has zoonotic importance. They said that this parasite is transmitted to herders by any means of contact causing stress and physical damage on the skin and it may create wounds.

## 4. Discussion

A total of 384 camels were examined for the presence of any type of external parasite and 368 (95.8%) of them were infested at least with one ectoparasite. Similarly, high ectoparasite infestations (100%) were reported by Aboma et al. [28] in camels slaughtered at Addis Ababa abattoir, Bekele [29] in southern Ethiopia, Al-Ani et al. [30] and Sharrif et al. [31] in Jordan, and around 92.7% reported by Lawal, Ameh, and Ahmed [32] in Nigeria. But the current prevalence is higher than that reported by Anwar and Khan [33] in Pakistan (57%) and Dia [34] in Burkina Faso. The higher ectoparasite prevalence recorded in this study could be due to continuous mixing and contact of camel herds at the salt trade line and watering points and inadequate veterinary services [19].

Risk factors such as sex, age, district, body condition score, and season were well thought out as influencing the occurrence and prevalence of ectoparasitisim in camels. But all risk factors have no significant statistical association (*p* > 0.05) with ectoparasitisim. Regardless of the different categories of risk factors, higher prevalence was recorded in each category and found with no statistical differences. This higher prevalence of ectoparasitic infestation of most camels regardless of the abovementioned risks could be due to cross infestation among camels because of continuous mixing of camel herds and limited chemotherapeutic and control efforts. Majority of the sampled camels (82.3%) were not in good body condition, but it was not a statistically significant association because camels may lose their body weight due to other factors such as poor husbandry and management practices, environmental stress, other concurrent diseases, and feed and water shortage [20].

The main ectoparasites recognized were ticks, lice, Sarcoptes mange, and nasal bot fly called *Cephalopina titillator*. Tick infestations (89.1%) followed by mange (36.7%) were the main ectoparasites found in the study area. In agreement with this study, a high prevalence of camel ticks (94%) was also reported by Ayele and Mohamed [35] in and around Dire Dawa, Zeleke and Bekele [36] in eastern Ethiopia and 100% by Aboma et al. [28] in camels slaughtered at Addis Ababa abattoir, Elghali and Hassan [37] in northern Sudan and Nourollahi Fard et al. [38] in southeast Iran, and around 91.2% by Lawal, Ameh, and Ahmed [32] in Nigeria. This higher prevalence could be due to limited acaricide availability and inaccessibility to veterinary services in the study area [19].

Similar to this study, camel mange is also reported by Feyera et al. [15] from Fafan zone, eastern Ethiopia. The recorded prevalence in this study is higher than the figures of Awol et al. [39], Dinka, Eyerusalem, and Yacob [40] and Lawal, Ameh, and Ahmed [32] who reported a prevalence of 16.7%, 10.7%, and 3.5% from Northern Ethiopia, Eastern Ethiopia, and Nigeria, respectively, and lower than a prevalence of 83% reported by Al-ani et al. [30] in Jordan. Variation in environment, accessibility to veterinary services, herd size, and other husbandry practices could be contributing factors for the differences. Sarcoptes was identified as the only mite species in all scrapings collected from suspected skin lesions. This observation is in agreement with reports by various authors [32, 39, 40].

The other camel external parasites identified were lice (23.2%), which is identified as the sucking lice of the order *Anoplurida* that was found on the head, neck, and withers of the sampled camels [41]. In addition, nasal bot fly *Cephalopina titillator* (17.7%) is also identified. Even though the larvae of the nasal bot are commonly found inside the pharyngeal cavities, it was collected while the camels are sneezing due to its irritative nature. About 68.2% infestation of nasal bot was previously reported by Aboma et al. [28] in camels slaughtered at Addis Ababa abattoir. In other reviews of reports from Africa and Asia, including the Middle East, the infestation rates varied between 47% and 100% [42]. About 46.7% infestation rate was found in camels in Iraq [43]. The highest incidence of larval infestation during the year was in January–March, the lowest in December. A survey in Saudi Arabia revealed that 32 out of 35 camels were infested [44]. Moreover, Fatani and Hilali [45] reported 52% of the camels were infested, peaking in February and September at Al-Asha abattoir in Saudi Arabia. The variation in the prevalence of this study and the aforementioned reports is the difference in method and season of sample collection.

In addition to single infestations, mixed infestations of tick with mange (30.2%), ticks with lice (19.8%), and ticks with nasal bot (16.0%) were recorded. This finding has shown that the prevalence of ticks as a single and mixed infestation was higher than the other camel ectoparasites. Moreover, around 1424 ticks were collected from the body of camels, in which 782 (54.9%) were male ticks to show the sex-based tick dynamics and its role in vector borne diseases transmission [27]. The tick genera identified in this study were *Amblyomma*, *Hyalomma*, *Rhipicephalus*, and *Boophilus* with an infestation intensity rate of 0.42, 1.96, 1.64, and 0.06, respectively. Even though it is not common to find *Boophilus* species in camels, but sometimes bovine *Boophilus* species can be found in camels with low prevalence rarely [41]. There are similar reports of tick genera by Zeleke and Bekele [36] in eastern Ethiopia, Ayele and Mohamed [35] in and around Dire Dawa, Ethiopia, Aboma et al. [28] in camels slaughtered at Addis Ababa abattoir, Lawal, Ameh, and Ahmed [32] in Nigeria, Elghali and Hassan [37] in northern Sudan, and Nourollahi Fard et al. [38] in southeast Iran.

The species level infestation intensity of ticks per camels showed that *Rhipicephalus pulchilus* (1.40) and *Hyalomma dromedarii* (1.18) had higher intensity rate as compared with the other species identified and *Boophilus decoloratus* (0.06) with relatively lower intensity. Similar infestation rates had been reported by [46] from Ethiopian Somali region and [10] from different parts of Ethiopia.

The cross-region salt trade in Aba'ala and Berahle using camels has been facilitating the transmission of all contagious diseases including manges and ticks due to mixing of camels coming from different areas [19]. In Dubti district, mixing of camels of different herds at watering and feeding points has facilitated the transmission of ectoparasites among them. The impact of camel parasitic infestation on camel productivity and health are due to loss of appetite, body condition loss and emaciation, wasting disease, milk drop, stress, tick worry and physical damage, morbidity, and mortality due to tick-borne diseases [11]. These conditions become serious especially in lactating she camels and young animals [11, 19].

The public health implication of mange is causing stress and physical damage to the skin of herders and it can be complicated with secondary bacterial infection and cause systemic diseases [12]. It is transmitted from the camels to the herders directly by physical contact or indirectly from the materials used to handle and manage camels.

## 5. Conclusion

The prevalence of external parasites in camels was recorded to be higher in the study area. Various groups of ticks, lice, Sarcoptes mange, and nasal bot were the identified ectoparasites that have been occurred in both sexes and in all age groups regardless of the body condition and origin of the camels. Higher prevalence of tick infestation evidenced as single and concurrent infestation with lice, mange, and nasal bot was recorded. This has been occurred in areas with salt trade by camels across the border and mixing of herds at the feeding and watering points. There was also a shortage of parasitic treatment due to inadequate drug supply in the area. Therefore, collaborative parasitic control and prevention measures have to be implemented from source and vehicles of transmission have to be altered by seasonal and programmed antiectoparasitic treatment and spray schemes.

## Figures and Tables

**Table 1:** Multivariable logistic regression analysis of risk factors associated with ectoparasitisim from Decemebr 2020 to June 2021.

Risk factor	Category	No. of examined⁣^∗^	Positive (%)	OR (95% CI)	*p* value
District	Aba'ala	230	221 (96.1)	—	
	Berahle	106	101 (95.3)	1.04 (0.21–5.04)	0.96
	Dubti	48	46 (95.8)	1.16 (0.21–6.41)	0.86

Sex	Male	146	143 (97.9)	—	
	Female	238	225 (94.5)	0.35 (0.10–1.25)	0.10

Age	≤ 4 years	173	165 (95.4)	—	
	> 4 years	211	203 (96.2)	1.27 (0.46–3.52)	0.64

Body condition score	Good	68	67 (98.5)	—	
	Fair	185	177 (95.7)	0.26 (0.03–2.14)	0.21
	Poor	131	124 (94.6)	0.84 (0.29–2.42)	0.75

Season	Dry	192	186 (96.9)	—	
	Rainy	192	182 (94.8)	0.59 (0.21–1.68)	0.32

⁣^∗^Total number examined was 384.

**Table 2:** Prevalence of major camel external parasites in the selected districts of Afar region from December 2020 to June 2021.

Ectoparasites	No of camel infested	Prevalence (%)
Tick	349	89.1
Mange	141	36.7
Lice	89	23.2
Nasal bot fly	68	17.7

**Table 3:** Prevalence of single and mixed camel ectoparasites in selected districts of Afar region from December 2020 to June 2021.

Type of infestation	Etiology	No. of camels infested	Prevalence (%)
Single	Tick only	106	28.8
	Mange only	11	3.0
	Lice only	5	1.4
	Nasal bot fly only	3	0.8

Mixed	Tick and mange	111	30.2
	Tick and lice	73	19.8
	Tick and nasal bot	59	16.0

Total		368	100

**Table 4:** Identified genera of camel ticks and their proportion and infestation intensity in selected districts of Afar region from December 2020 to June 2021.

Tick genera	Number of ticks		Total	Ratio	Infestation intensity⁣^∗^ (*n* = 349)
	Female	Male		Female to male	
*Amblyomma*	54	92	146	0.58	0.42
*Hyalomma*	260	423	683	0.61	1.96
*Rhipicephalus*	322	250	572	1.29	1.64
*Boophilus*	6	17	23	0.35	0.06
Total	642	782	1424	0.82	4.08

⁣^∗^(ticks/camel), *n* = 349 is the total number of camels positive for tick infestation.

**Table 5:** Infestation intensity and relative prevalence of camel tick species identified in selected districts of Afar region from December 2020 to June 2021.

Tick species	Number of ticks	Relative prevalence (%) (*n* = 1424)	Infestation intensity (*n* = 349)
*Amblyomma variegatum*	62	4.35	0.18
*Amblyomma lepidium*	48	3.37	0.14
*Amblyomma gemaa*	36	2.53	0.10
*Hyalomma dromedarii*	413	29.0	1.18
*Hyalomma truncatum*	270	18.96	0.77
*Rhipicephalus pulchilus*	489	34.34	1.40
*Rhipicephalus evertsi*	83	5.83	0.24
*Boophilus decoloratus*	23	1.62	0.06

*Note: n* = 1424 is the total number of ticks collected from the infested camels and *n* = 349 is the total number of camels positive for tick infestation.

## Data Availability

The data that support the findings of this study are available from the corresponding author upon reasonable request.
